# RNA Tailing by Nucleotidyltransferases in Plants: Mechanisms, Functions, and Biological Significance

**DOI:** 10.3390/plants15060925

**Published:** 2026-03-17

**Authors:** Xintong Xu, Xinwen Qing, Xiaoli Peng, Xiangze Chen, Tengbo Huang, Beixin Mo, Yongbing Ren

**Affiliations:** 1 Guangdong Provincial Key Laboratory for Plant Epigenetics, College of Life Sciences and Oceanography, Shenzhen University, Shenzhen 518060, China; xxt19950625@163.com (X.X.); coratheone@163.com (X.Q.); pengxiaoli@szu.edu.cn (X.P.); chenxz@szu.edu.cn (X.C.); tengbohuang@szu.edu.cn (T.H.); 2 Ganzhou Sub-Center of National Vegetable Quality Standards Center, Ganzhou 341000, China

**Keywords:** agronomic traits, gene expression, nucleotidyltransferase protein (NTP), plant antiviral immunity, plant development, RNA stability, RNA tailing, stress response

## Abstract

RNA tailing, the non-templated addition of nucleotides to RNA 3′ ends, is a conserved post-transcriptional modification that plays a critical role in regulating RNA metabolism. In plants, this process is primarily mediated by nucleotidyltransferase proteins (NTPs). In this review, we analyze current knowledge of plant NTPs by integrating evidence from genetic, biochemical, and phylogenetic analyses of the gene-family across model plants and crops. We summarize the composition and evolutionary diversification of the plant NTP gene family, with emphasis on lineage-specific expansion and conservation patterns. Using *Arabidopsis thaliana* as a reference framework, we then describe the molecular roles of NTPs in the tailing of distinct RNA classes, emphasizing how tail type and length confer context-dependent regulatory outcomes including stabilization versus degradation and processing/maturation versus clearance. We further examine the determinants of substrate choice, focusing on RNA type, terminal structure, and subcellular localization. Finally, we discuss the biological functions of NTP-mediated RNA tailing in plants, linking RNA tailing to development, stress responses, antiviral immunity, and agronomic traits in crops. We conclude by outlining key mechanistic and physiological challenges that define future directions for understanding and harnessing NTP-mediated RNA regulation. Collectively, this review provides an integrated framework for understanding how RNA tailing by NTPs shapes plant RNA metabolism and biological fitness.

## 1. Introduction

RNA metabolism is central to the regulation of plant gene expression, coordinating plant development and stress acclimation [1]. Post-transcriptional processes, including epitranscriptomic modification, RNA processing, stability control, and regulated decay, jointly define the lifespan and functional output of transcripts, thereby shaping cellular expression patterns and adaptive responses [1,2]. Within this continuum, the non-templated addition of nucleotides to the 3′-end of RNA (RNA tailing) is a conserved and widespread phenomenon that modulates RNA metabolism, primarily by tuning RNA stability, and integrates maturation, surveillance, and turnover [3,4,5].

NTPs, also known as terminal nucleotide transferases (TNTs), are a class of template-independent RNA polymerase that add nucleotides to the 3′-ends of RNA, thereby shaping the end heterogeneity of RNAs, and directly affecting their stability, translation efficiency, and degradation pathways [3,6,7]. NTPs share a conserved Pol-β–like catalytic core and contain enzymes capable of attaching nucleotides to proteins, antibiotics, or RNAs, but diversify in accessory domains and interacting partners, yielding distinct activities and substrate specificities across different RNA classes [8]. Non-template tailing of small RNA precursors, messenger RNAs (mRNAs), or mRNA cleavage products by NTPs has been found in plants [9,10,11,12], humans [13], yeast [14], *C. elegans* [15], and fungi [16], where it mainly affects the stability of these diverse RNA substrates.

The study of NTPs in eukaryotes is now well established. In mammals, TUT1, TUT4 (ZCCHC11), and TUT7 (ZCCHC6) represent the major uridylation enzymes, whereas GLD-2 (PAPD4/TENT2), TENT4A/B (PAPD7/5), the TENT5 family, and MTPAP function predominantly as adenylation enzymes [15,17,18,19,20,21]. TUT1 supports U6 snRNA 3′-end maturation by adding oligo(U) tails, which are subsequently trimmed by USB1 to generate the mature 3′ end and promote LSm2–8 binding [22,23]. TUT4/7 control pre-miRNA processing: under Lin28-dependent conditions, they oligouridylate pre-let-7, block Dicer cleavage, and recruit DIS3L2 for degradation, thereby suppressing let-7 biogenesis [18,24,25]. In the absence of Lin28, however, TUT4/7 can mono-uridylate specific pre-miRNAs to restore the optimal 3′ overhang and enhance Dicer processing, revealing context-dependent bidirectional activity [19,26]. On the adenylation side, GLD-2 stabilizes miR-122 through mono-adenylation [27], while TENT4A/B introduce mixed A/G tails at mRNA 3′-ends to slow deadenylation and extend transcript longevity [28]. Similar principles apply in other eukaryotes: In *Schizosaccharomyces pombe*, the noncanonical nucleotidyltransferases Cid1/Cid16 catalyze 3′ uridylation of RNA substrates and are implicated in mRNA turnover [4,14,21,29,30]; *Chlamydomonas* MUT68 promotes exosome-dependent turnover of RISC-cleaved transcripts and contributes to small-RNA decay [17,31]; *Drosophila* Tailor restricts mirtron-derived pre-miRNA maturation, and *C. elegans* CDE-1 uridylates CSR-1–bound 22G-RNAs [15,20,32,33]. In budding yeast, TRAMP (TRF4/5) oligoadenylates aberrant nuclear RNAs to target them to the exosome [34,35,36].

Taken together, studies across diverse species demonstrate that NTPs, by writing specific uridylation or adenylation signatures at RNA 3′ ends, finely regulate the processing and homeostasis of pre-miRNAs, miRNAs, siRNAs and the decapping-coupled exonucleolytic decay of mRNAs. Reprogramming the chemical environment and tail composition at the 3′ terminus thus constitutes a key regulatory layer that links RNA processing, stability, translation, and clearance in eukaryotic RNA metabolism.

Although 3′-end tailing is broadly conserved across eukaryotes, its regulatory logic differs substantially among plants, animals, and yeast, particularly in small-RNA pathways. In plants, HUA ENHANCER 1 (HEN1) is a 2′-O-methyltransferase that methylates small RNAs at their 3′ termini, and this modification plays a central role in safeguarding miRNAs and siRNAs from tailing-mediated degradation [37,38]. The identification of HEN1 laid the foundation for uncovering the enzymes responsible for modifying unmethylated small RNAs. In this plant-specific framework, NTP-catalyzed tailing often functions as an opposing layer of regulation: when methylation is absent or compromised, small RNAs become more susceptible to 3′ tailing, which can shift their fate toward altered processing outcome or accelerated turnover. By contrast, animals and yeast lack an analogous, broadly deployed HEN1-dependent protection mechanism for their small-RNA populations, and 3′ tailing is more tightly integrated with organism-specific RNA surveillance and decay networks. In 2012, forward-genetic screens in *Arabidopsis* identified HEN1 SUPPRESSOR1 (HESO1, NTP1) as the major NTP that uridylates unmethylated miRNAs in *hen1* background, thereby promoting their degradation and partially rescuing the *hen1* phenotype [39,40]. Subsequent biochemical and genetic analyses further found URIDYLYL TRANSFERASE 1 (URT1, NTP3) as an additional TUTase involved in 3′ uridylation of miRNAs, with distinct processivity and substrate preferences compared with HESO1 [9,41,42]. Although both HESO1 and URT1 mediate non-templated uridylation at RNA 3′ ends, they exhibit significant differences in substrate specificity and catalytic processivity. In recent years, rapid advances in high-throughput RNA sequencing technologies have enabled the progressive functional characterization of additional plant NTPs.

During the preparation of this review, a related article by Chen et al. (2024) summarized recent progress in plant RNA tailing with an emphasis on uridylation-centered RNA metabolism [43]. To complement and extend this work, our review provides a broader and more integrative synthesis of plant NTPs across species and RNA pathways. Here, based on available data from reported plant species, we systematically characterize the composition and evolutionary relationships of the NTP family across plants. Using *Arabidopsis thaliana* as a reference, we focus on gene structural features and catalytic mechanisms of its ten NTP members, revealing significant conservation in core domains and nucleotides transfer activities within this family. Based on this foundation, we further elucidate the high specificity exhibited by individual members in substrate recognition and modification patterns, as well as the intricate division of labor and cooperative mechanisms operating across multiple RNA metabolic pathways.

## 2. Compositions of the Plant NTP Gene Family

In recent years, studies from multiple laboratories have identified numerous NTPs and illustrated their specialized roles across diverse plant species [43,44,45,46,47]. In *Arabidopsis thaliana*, HESO1 and URT1 have been well characterized as key enzymes involved in RNA 3′-end tailing [39,40,43,48]. Homologous or functionally related proteins have also been identified in major crops such as rice [45,47], maize [44], soybean [46] and wheat [49], reflecting the evolutionary conservation of these enzymes, as well as the species-specific diversification of their biological functions. Collectively, these findings underscore the central role of NTPs in plant RNA metabolism, linking post-transcriptional RNA modifications to broader aspects of plant growth, productivity, stress responses, and disease resistance.

### 2.1. Gene Family Size and Phylogenetic Organization Across Species

**Figure 1:**
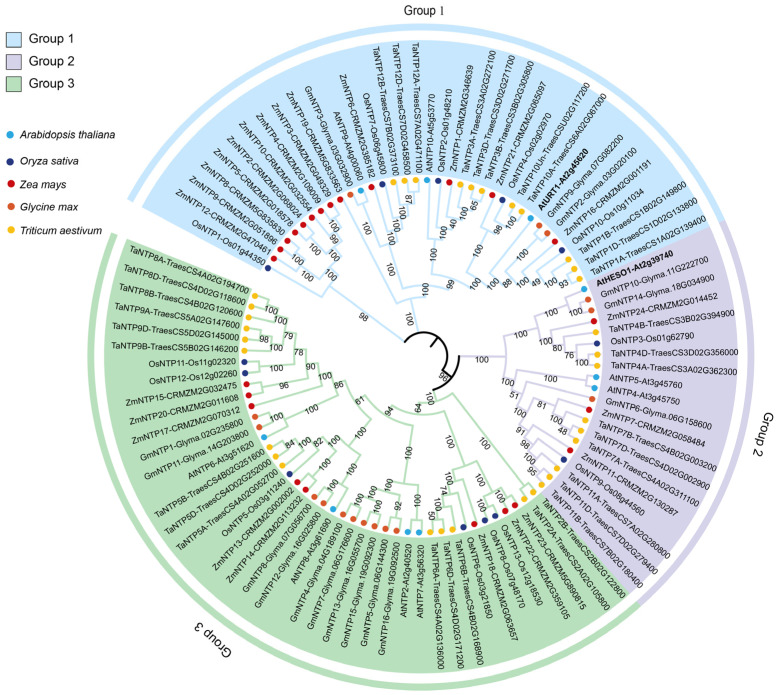
Phylogenetic analysis of NTPs in *Arabidopsis thaliana*, *Oryza sativa*, *Zea mays*, *Glycine max* and *Triticum aestivum*. Protein sequences were aligned using ClustalW, and the phylogenetic tree was constructed in MEGA12 using the neighbor-joining method with p-distance. Bootstrap support values were calculated from 1000 replicates and are shown at the nodes. Values < 50% were omitted, and the displayed bootstrap values are grouped into three ranges (50–69%, 70–89%, and ≥90%) to facilitate readability. NTP members from each species were assigned to subfamilies based on their clustering with *Arabidopsis* reference clades. For bread wheat (*Triticum aestivum*), TaNTP genes are labeled with their subgenome origin (A, B, or D) when available, and paralogs or homeologs were classified based on their positions within the corresponding subfamily clades. NTPs were categorized into three distinct groups (G1, G2, and G3), which are indicated in blue, purple and green, respectively, in the polygenetic tree.

With the rapid advancement of plant genomics, the genomic distribution of NTP family members has also been systematically characterized. *Arabidopsis* encodes 10 NTPs, whereas major crops such as rice, maize, soybean and wheat harbor 13 [45,47], 24 [44], 16 [46] and 34 [49] members, respectively. This expansion suggests that RNA tailing functions have diversified to meet species-specific regulatory demands [8,43,44,45,46,49] (Table 1; Supplementary Materials, Table S1). Phylogenetic analyses of *Arabidopsis*, rice, maize and soybean indicate that these genes can be divided into three major evolutionary clades (Group I–III; Figure 1). In our phylogeny, Group I members, represented by Arabidopsis URT1, and Group II members, represented by *Arabidopsis* HESO1, are highly conserved across species, implying a conserved role for RNA 3′-end modification in plant species.

However, the significant expansion of NTP members in maize, soybean and wheat reflects lineage-specific duplication and diversification. In soybean, NTP genes are often retained as homologous pairs, consistent with its history of whole-genome duplication (WGD), whereas maize NTPs show expansion across multiple phylogenetic branches, suggesting more complex subfunctionalization patterns. In hexaploid wheat, the increased NTP copy number largely corresponds to homoeolog retention across the A, B, and D subgenomes, with many TaNTPs forming triads distributed across the three major clades. Lineage-specific genome histories provide a plausible evolutionary basis for this expansion and diversification. Soybean (*Glycine max*) is a palaeopolyploid that experienced at least two rounds of whole-genome duplication (∼59 and ∼13 million years ago), resulting in a highly duplicated genome in which a large fraction of genes are retained as duplicates [50]. These WGD-derived duplicates provide an evolutionary substrate for subfunctionalization and neofunctionalization, helping to explain lineage-specific expansion patterns observed in comparative NTP phylogenies [50]. Bread wheat (*Triticum aestivum*) is a relatively young allohexaploid (AABBDD) formed through hybridization between a domesticated tetraploid (AABB) and the diploid donor *Aegilops tauschii* (DD), resulting in three retained subgenomes (A/B/D) and extensive homoeologous gene sets. Such polyploidization, together with differential retention or loss among subgenomes can promote clade-level copy-number expansion and provide opportunities for expression divergence and functional differentiation [51,52]. Overall, this expansion not only reflects the evolutionary diversification of NTPs among plant lineages, but also suggests potential functional divergence in regulating developmental processes and stress responses.

### 2.2. Functional Insights from Crop Genetics

Notably, crop genetics research has provided direct evidence for the functional roles of NTP family members. In rice, genome-wide association analysis (GWAS) combined with functional validation showed that the natural variation *LOC_Os01g62790*, the *Arabidopsis HESO1* homolog, significantly influences flowering time [45]. This study represents the first direct link between RNA tailing enzymes and key agronomic traits in crops, demonstrating that NTP family members function not only at the molecular level but also exert a decisive influence on trait formation. In hexaploid wheat, haplotype variation in TaNTP6A/B/D is significantly associated with thousand-kernel weight (TKW), and TaNTP6 genes show grain-enriched expression patterns, implicating NTP-mediated RNA tailing as a potential regulatory layer contributing to yield-related traits in cereals [49]. Overall, these studies connect NTP function to agronomically important traits across monocot crops, expanding their relevance from molecular RNA metabolism to crop performance.

### 2.3. Gene Family Composition and Evolutionary Architecture in Arabidopsis

**Figure 2:**
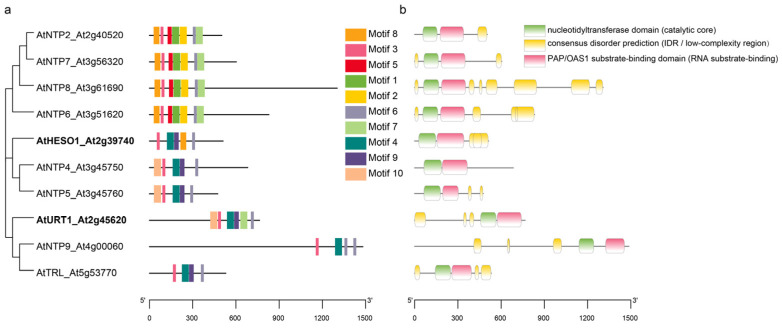
Conserved motifs and domain organization of NTPs in *Arabidopsis*. (**a**) Phylogeny-guided summary of conserved protein motifs (Motifs 1–10) across *Arabidopsis* NTPs. Motifs were identified using MEME (top 10 motifs). Representative functional anchors are highlighted. (**b**) Predicted domain architecture of *Arabidopsis* NTPs based on InterPro annotations. Green indicates the nucleotidyltransferase (NTase) catalytic core, pink indicates the PAP/OAS1-like substrate-binding domain, and yellow indicates predicted intrinsically disordered regions (IDRs). The diverse distribution of IDRs among paralogs may contribute to functional diversification and context-dependent regulation in RNA metabolism pathways.

The ten NTP members in *Arabidopsis* (HESO1/NTP1, URT1/NTP3, NTP2, NTP4, NTP5, NTP6, NTP7, NTP8, NTP9 and TRF4/5-LIKE (TRL/NTP10)) exhibit both conserved structural frameworks and remarkable diversity in regulatory features, which determine their substrate preferences. From an evolutionary perspective, these ten NTPs can be divided into three major clades (Figure 2a). The first group includes NTP2, NTP7, NTP6, and NTP8, which cluster together in phylogenetic tree, with highly similar motif architectures, demonstrating evolutionary conservation. Although functional studies of this group remain limited, sequence and structural evidence suggest partial redundancy in their activities. For example, NTP6 and NTP7 have been reported to cytidylate certain pre-miRNAs [11]. The second group consists of HESO1, NTP4, and NTP5. HESO1 is the best-characterized member of this clade and has been established as a typical TUTase that plays a key role in small RNA tailing and degradation, directly regulating miRNA stability [39,40,41,42]. In contrast, NTP4 and NTP5 exhibit highly similar domain architectures and motif arrangement, implying overlapping or complementary substrate preferences. The third clade comprises URT1, NTP9, and NTP10, which form a distinct subclade. URT1 plays a crucial role in uridylating oligoadenylated mRNAs and shaping cytoplasmic mRNA decay pathways [9,10,53], and it functionally complements HESO1 in regulating small RNA stability and degradation processes [39,40,41,42]. NTP9 and TRL/NTP10 share structural and motif similarities with URT1, suggesting a functional bias toward specific RNA regulation. Collectively, this classification highlights both conservation and diversification among *Arabidopsis* NTPs, providing an evolutionary framework for further elucidating their substrate specificity and biological functions.

### 2.4. Conserved and Divergent Structural Features

All NTPs contain the conserved nucleotidyltransferase domain (cd05402), which constitutes the catalytic core responsible for nucleotide tailing reactions. This domain is highly conserved across family members, ensuring fundamental enzymatic stability [8,54]. In addition, the Poly(A) polymerase/2′–5′-oligoadenylate synthetase 1 (PAP/OAS1) substrate-binding domain (SSF81631) is universally present, supporting stable RNA-active site interactions [8,55,56]. It is worth noting that several NTP members commonly possess intrinsically disordered regions (IDRs) of significant length. This structural feature implies additional regulatory complexity beyond traditional enzyme catalytic functions, potentially enabling dynamic protein-RNA or protein–protein interactions (Figure 2b).

In summary, the plant NTP family preserves highly conserved core functions in RNA terminal modification while undergoing lineage-specific expansion and functional diversification. These dual characteristics highlight both functional conservation and adaptability within the family and offer a molecular framework for understanding how RNA metabolism contribute to crop adaptability and trait formation. They also provide potential targets and new research directions for crop molecular design and breeding.

## 3. Molecular Roles of NTPs in the Tailing of RNA Classes

NTPs are essential enzymes in multiple RNA metabolic pathways. Unlike canonical poly(A) polymerases, which primarily adenosine tails, NTPs catalyze various non-canonical tailing reactions, including the addition of adenosine (A), uridine (U), cytidine (C), and, in some contexts, guanosine (G). Among these, uridyltion represents the most common modification conferred by plant NTPs [5,8,43]. The major RNA classes targeted by *Arabidopsis* NTPs are summarized in Table 2.

### 3.1. Tailing of microRNAs and miRNA*

In plants, mature miRNAs and their miRNA* counterparts are safeguarded by HEN1-mediated 2′-O-methylation at their 3′ ends, a modification that prevents both exonucleolytic trimming and non-templated tailing, thereby stabilizing the duplex [37,57]. In *hen1* mutants, this protective methylation is absent. Under these conditions, HESO1 efficiently adds one or multiple uridines to exposed 3′-ends of miRNAs/miRNA*, generating oligouridylated species that are rapidly destabilized and degraded [39,40]. Consistent with this enzymatic activity, *hen1 heso1* double mutants exhibit a substantial restoration of miRNA abundance [39,40]. URT1, a paralog of HESO1, can also uridylate unmethylated miRNAs/miRNA*, but it generally shows lower processivity and a narrower substrate range, preferentially targeting miRNAs ending in adenosine [41,42,43]. However, *heso1 urt1 hen1* triple mutants exhibit even greater recovery of miRNA levels than *heso1 hen1* double mutants, revealing a cooperative yet hierarchical relationship between the two enzymes [41,42]. Together, HESO1 and URT1 constitute the canonical degradation-oriented pathway for unmethylated miRNAs in plants.

**Figure 3:**
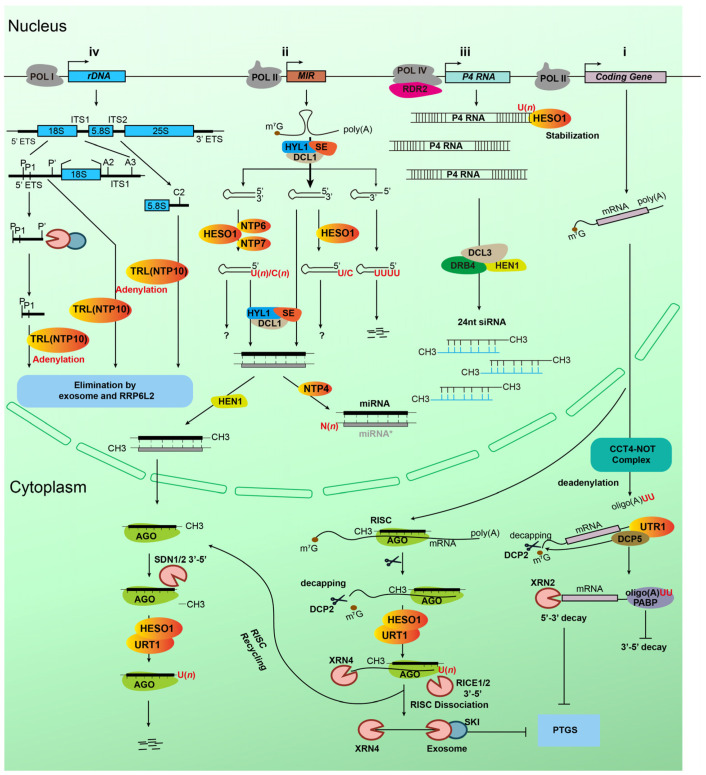
Diagram of biological roles of NTPs in RNA metabolism regulation in plants. NTP enzymes catalyze non-templated nucleotide addition (tailing) on diverse RNA substrates and function as master regulators of RNA metabolism. Their major functions include: (**i**) regulating mRNA stability and degradation through uridylation; (**ii**) controlling sRNA biogenesis, modification, and stability by marking molecules for degradation or influencing precursor processing; (**iii**) stabilizing P4 RNAs through uridylation in the RNA-directed DNA methylation (RdDM) pathway; (**iv**) maintaining ribosomal RNA quality via adenylation-mediated turnover of precursors. PTGS, post-transcriptional gene silencing. Other components are labeled as indicated in the figure.

During miRNA biogenesis, after DCL1 processing, the miRNA/miRNA* duplex undergoes strand selection: the guide strand is loaded into AGO1 to form RISC, whereas the passenger strand (miRNA*), is typically subjected to degradation [37,57]. NTP-mediated tailing can introduce asymmetric modifications within the duplex, thereby influencing the fate of each strand [58,59]. For example, NTP4 monouridylates a subset of miRNA* strands (e.g., miR156*) without affecting the corresponding guide strand, resulting in increased accumulation of the guide miRNA and reduced levels of miR156*. This asymmetry likely reflects duplex structural preferences, as NTP4 prefers substrates with a 2-nt 3′ overhang and competes with HEN1 for binding. Consistently, *ntp4* mutants show altered global miRNA profiles, indicating that the selective miRNA* modification by NTP4 contributes to miRNA stability regulation [58] (Figure 3).

### 3.2. Tailing of Endogenous siRNAs

Plant small RNAs (sRNAs), typically 20–24 nucleotides (nt) in length, mainly include microRNAs (miRNAs) and small interfering RNAs (siRNAs). In general, miRNA/miRNA* duplexes often contain mismatches or bulges, whereas siRNA duplexes show higher complementarity, reflecting their distinct biogenesis pathways [60,61,62]. In principle, any small RNA lacking HEN1-dependent 2′-O-methylation becomes susceptible to 3′-end uridylation by NTPs, and experimental evidence confirms that this applies not only to miRNAs but also to endogenous 21-nt siRNAs [39,42,43,63]. Early deep-sequencing analyses revealed widespread 3′ truncation and tailing among plant small RNAs, with uridylation levels strongly altered when 2′-O-methyl protection is compromised or tailing enzymes are genetically perturbed [59]. The identification of HESO1 as the major uridylase targeting unmethylated small RNAs provided a mechanistic explanation for this observation [39,40]. Genome-wide analyses of *heso1*, *urt1* and *heso1 urt1* double mutants further demonstrated that both miRNAs and endogenous siRNAs acquire 3′ U-tails when HESO1 and URT1 activity is reduced or absent, indicating that NTP-mediated tailing broadly affects multiple small RNA populations, including 21-, 22-, and 24-nt species [39,42,59,64]. Thus, methylated siRNAs enter the same tailing-dependent degradation pathway as unmethylated miRNAs.

### 3.3. Tailing of mRNA Cleavage Fragments

Uridylation plays an important role in elimination 5′ fragments produced by RISC mediated cleavage [43,48,50]. HESO1 and URT1 act as primary initiators of the RISC recycling pathway by uridylating cleavage fragments [48,65,66]. Following miRNA-guided cleavage, the resulting 5′ mRNA fragment is primarily uridylated by HESO1, with URT1 contributing secondarily. The uridylated 5′ fragment is then recognized and degraded by RISC-interacting exonuclease (RICE1/2), which facilitates AGO recycling and prevents the generation of aberrant secondary siRNA [65,66,67].

Uridylation is tightly coupled with mRNA quality control mechanisms and plays a critical role in suppressing spurious post-transcriptional gene silencing (PTGS) [42,43,53,66]. In *Arabidopsis thaliana*, simultaneous impairment of uridylation pathway and 3′→5′ exonuclease decay pathway leads to aberrant accumulation of mRNA cleavage fragments derived from TRANSKETOLASE 1 (TKL1), a key gene in the Calvin cycle [68]. These fragments are recognized by RNA-dependent RNA polymerase 6 (RDR6) and converted into double-stranded RNA (dsRNA), leading to large amounts of illegitimate 21-nt siRNAs, which in turn trigger PTGS and cause developmental defects such as leaf chlorosis. Notably, the molecular and physiological abnormalities can be substantially rescued by dysfunction of RDR6, confirming the importance of the “uridylation–mRNA decay-PTGS suppression” axis in maintaining the homeostasis of endogenous gene expression [43,50,67,69].

Genetic analyses strongly support this model. In *urt1*, *heso1*, and especially *urt1 heso1* double mutant, both 5′ and 3′ cleavage fragments accumulate to higher levels, and many miRNA targets produce ectopic secondary siRNAs due to leakage into the RDR6 pathway [42,43,53,66]. These phenotypes become even more pronounced in *urt1 ski2* mutants, where defects in both uridylation and exosome-mediated 3′→5′ decay synergize, leading to massive buildup of cleavage intermediates and widespread illegitimate 21-nt siRNA production [53]. Importantly, these phenotypes are suppressed by RDR6 dysfunction, confirming that the developmental defects originate from inappropriate siRNA amplification caused by inefficient clearance of miRNA-cleaved fragments [53]. Accumulating evidence indicates that uridylation also actively promotes the turnover of miRNA-derived 5′ cleavage fragments through the 5′→3′ RNA decay pathway [48,70]. By marking these fragments for rapid removal, uridylation facilitates their dissociation from the RISC complex and targets them for exonucleolytic degradation. This function is exemplified in *Arabidopsis*, where the uridylated 5′ fragment of *MYB33* accumulates to disproportionately higher levels when normal decay pathways are compromised, revealing that uridylation not only marks 5′ fragments for degradation but also shapes the characteristic truncation patterns observed among RISC-generated 5′ products [43,48,70].

### 3.4. Tailing of Intact mRNA

In plants, the stability of mRNA is mainly maintained by its 5′ cap and 3′ poly (A) tail [71]. Decapping and deadenylation are the key steps in mRNA degradation. Uridylation at the 3′ end of poly(A) tail also plays an important role in mRNA degradation pathway [9,10,43,53]. In *Arabidopsis,* 3′ terminal uridylation of mRNA is mainly catalyzed by URT1 and HESO1. This modification serves multiple roles in mRNA metabolism, primarily by regulating mRNA stability through maintaining poly(A) integrity and directing mRNAs into appropriate degradation pathways as part of RNA surveillance systems [10,69].

Although uridylation has long been associated with RNA decay, accumulating evidence indicates that, for intact mRNAs, its primary role is not to trigger degradation but to prevent excessive deadenylation and channel transcripts into the appropriate decay pathway [9,10,53,69]. URT1 is the major TUTase responsible for this activity, preferentially uridylating oligoadenylated mRNAs with short oligo(A) tails (10–25 nt) by adding one or two uridines [9,10]. These short U-tails help restore an optimal tail length of ~16 nt, promote rebinding of poly(A)-binding proteins (PABPs), and prevent transcripts from entering aberrant deadenylation-driven decay [9,10]. In addition, URT1 physically interacts with the decapping activator DCP5, linking the CCR4–NOT deadenylase complex to the decapping machinery and thereby channeling oligoadenylated transcripts into the XRN4-mediated 5′→3′ decay pathway [53]. Through this coordinated mechanism, URT1-mediated uridylation maintains poly(A) tail integrity and ensures proper mRNA turnover, acting as a central component of RNA surveillance [10,43,53]. Another study found that URT1 can uridylate, but does not shape, poly(A) tails in a microRNA-independent manner. This further indicates the role of URT1 in regulating intact mRNA with long poly(A) tails [68].

### 3.5. Tailing of rRNA Precursors

In plant cells, conventional mRNA polyadenylation is mainly catalyzed by canonical poly(A) polymerases (cPAPs), which ensure the stability and translation efficiency of transcripts [72,73]. However, certain members of the NTP family in *Arabidopsis* perform unconventional adenylation functions. For example, TRL/NTP10 adenylates the 3′ ends of rRNA precursors, including those of 18S and 5.8S rRNAs, providing a recognition and degradation signal that facilitates the recruitment of the exosome complex, thereby promoting targeted RNA turnover and contributing to ribosomal RNA quality control [74]. In addition, uridylation may also play a role in rRNA metabolism, but how uridylation affects the biosynthesis process of rRNA precursors remain unclear and requires further investigation [74].

### 3.6. Tailing of miRNA Precursors

In plants miRNA biogenesis, pre-miRNAs are intermediates produced by the microprocessor (DCL1/HYL1/SE) from hairpin structured pri-miRNAs [57,75,76]. Their 3′ ends typically bear a canonical 2-nt overhang before entering the subsequent processing step [57,77]. Systematic 3′ RACE-seq analyses show that nearly all detectable pre-miRNAs undergo some degree of 3′ non-templated tailing, most commonly single uridylation or cytidylation [11].

HESO1 is the predominant NTP responsible for pre-miRNA uridylation. In vitro, it uridylates substrates bearing correct 2-nt overhangs as well as multiple mis-end configurations, while in vivo it mediates the majority of pre-miRNA uridylation [11]. Tailing at exact endpoints is nearly abolished in *heso1* mutants, underscoring the central role of HESO1 in adding single nucleotides to intact precursors [11]. Additionally, NTP6 and NTP7 contribute to pre-miRNA cytidylation [11]. NTP-mediated pre-miRNA modification can be divided into three functional types: First, Occasional single nucleotide addition. HESO1 occasionally appends a single uridine or cytidine to intact pre-miRNAs, though the biological significance remains unclear. Second, Repair of abnormal ends. HESO1 together with NTP6 and NTP7, add U or C to abnormally trimmed pre-miRNAs to re-establish the canonical 3′ 2-nt overhang structure, likely to recruit renewed DCL1 processing. Third, Degradation of misprocessed precursors. HESO1 adds oligouridine tails to severely mis-processed pre-miRNAs, marking them for degradation (Figure 3).

### 3.7. Tailing of P4RNAs in the RdDM Pathway

In plant epigenetic regulation, the RNA-directed DNA methylation (RdDM) pathway is an important mechanism for maintaining genome integrity and establishing transcriptional gene silencing [78,79]. The core effectors of this pathway are 24-nt siRNAs, which are processed from double-stranded precursor, known as polymerase IV RNAs (P4RNAs). The biogenesis of canonical P4RNA is orchestrated by the coordinated actions of Pol IV and RDR2 [80,81], followed by precise cleavage by DICER-LIKE 3 (DCL3) to generate mature 24-nt siRNAs that guide DNA methylation. Multiple independent studies have reported non-templated nucleotide additions at the 3′-termini of P4RNAs [64,81,82]. Our previous study identified HESO1 as the core enzyme responsible for uridylation on P4RNA, catalyzing mono-uridylation, di-uridylation, and mixed nucleotide tails containing uridine (such as CU and UC). In this process, HESO1 exhibits strong substrate preference, preferentially adding uridine to P4RNAs ending in adenosine or uridine, while displaying minimal activity toward RNAs terminating in cytidine or guanosine. Dysfunction of HESO1 resulted in a substantial reduction in P4RNA uridylation levels, which subsequently leads to decreased stability of P4RNAs [12] (Figure 3).

**Table 2:** Functional overview of *Arabidopsis* NTP members, highlighting their localization, catalytic specificity, major RNA targets, and roles in RNA metabolism.

Gene	TAIR Locus	Localization	Putative Type	Main Substrates	Key Functions	Reference
HESO1 (NTP1)	AT2G39740	Cytoplasm and nucleus	uridylation	miRNA	Degradation	[39,40]
			uridylation/ cytidylation	Pre-miRNA	Processing/ Degradation	[11]
			uridylation	siRNA (het-siRNA)	Degradation	[39,42]
			uridylation	P4RNA	Enhance stability	[12]
			uridylation	mRNA?	Degradation/ Prevent deadenylation	[8]
			uridylation	5′ RISC-cleavage fragment	Degradation	[48,65,66]
			uridylation	Viral RNA	Degradation/ Enhance stability?	[83,84]
URT1 (NTP3)	AT2G45620	Cytoplasm (p-bodies and stress granules)	uridylation	mRNA	Degradation/ Prevent deadenylation	[9,10]
			uridylation	5′ RISC-cleavage fragment	Degradation	[48,65,66]
			uridylation	miRNA	Degradation	[41,42]
			uridylation	Viral RNA	Degradation/Enhance stability?	[83,84]
NTP2	AT2G40520	Unclear	Nucleotidyltrans-ferase family (paralog)	Unclear	Unclear	
NTP4	AT3G45750	nucleus	uridylation	miRNA/miRNA*	Enhance miRNA stability	[58]
NTP5	AT3G45760	Unclear	Nucleotidyltrans-ferase family (paralog)	Unclear	Unclear	
NTP6	AT3G51620	Unclear	cytidylation	Pre-miRNA	Unclear	[11]
NTP7	AT3G56320	Unclear	cytidylation	Pre-miRNA	Unclear	[11]
NTP8	AT3G61690	P-bodies	Nucleotidyltrans-ferase family (paralog)	Unclear	Unclear	[85]
NTP9 (MEE44)	AT4G00060	Unclear	Nucleotidyltrans-ferase family (paralog)	Unclear	Unclear	
TRL (NTP10)	AT5G53770	nucleolar	adenylation	Pre-rRNA	Degradation	[74]

## 4. Determinants and Regulation of Substrate Choice

### 4.1. Structural Architecture as a Basis for Selectivity

Notably, the retention of the typical NTP core catalytic domain coupled with the PAP/OAS1-like substrate-binding domains in all members, strongly suggests a conserved substrate recognition mechanism within this family. Variations in the organization, sequence features, and combinatorial arrangements of these structural modules are likely to underlie the substrate selectivity and unique biochemical activities of individual NTPs, ultimately driving their functional diversification (Figure 2 and Figure 3; Table 2).

*Arabidopsis* NTPs exhibit pronounced substrate specificity in RNA metabolism. This specificity operates at multiple levels, encompassing not only different RNA types but also fine scale preference for terminal nucleotide identity and RNA structural features, together forming a multilayered, finely tuned RNA modification network. (Table 2). Individual members show clear differentiation in substrate preference: For example, HESO1 tends to act on RNAs that end in uridine or adenosine and is particularly effective at adding oligo(U) tail(s) to unmethylated miRNA to guide it into the degradation pathway [39,40,41,42,86]; However, URT1 prefers to modify RNAs that end in adenosine, usually adding only 1–2 uridine residues, a modification that serves a protective role by preventing excessive deadenylation and degradation, playing a key role in maintaining RNA metabolic homeostasis [9,10,53,87]. Additionally, the activity of TRL/NTP10 is specialized for precursor rRNA, where its adenosylation modification promotes the degradation and clearance of pre-rRNAs, performing strict RNA quality monitoring function [74]. NTP6 and NTP7 act coordinately on pre-miRNAs and are responsible for cytidine addition [11].

Substrate selection is determined by both RNA secondary structure and terminal nucleotide composition. In the absence of HEN1-mediated methylation protection, HESO1 and URT1 act on un-methylated miRNAs in a coordinated and sequential manner: URT1 typically initiates monouridylation, generating U-terminated intermediates that serve as preferred substrates for HESO1, which then extends oligo U tail to trigger miRNA degradation [37,39,40,63,86]. This explains why knockout of HESO1 in *hen1* mutants significantly alleviates phenotypic defects, whereas knockout of URT1 does not [8,41,42,86]. Additionally, NTP4 exhibits “asymmetric modification” capabilities toward miRNA/miRNA* duplexes, selectively modifying only one strand to regulate small RNA stability and activity [58].

### 4.2. Subcellular Localization as a Determinant of Access to Substrates

The subcellular localization patterns of NTPs plays a crucial role in determining their access to specific RNA substrates and influence their functional activities (Table 2). For example, HESO1 is localized to both the nucleus and cytoplasm [39,40], reflecting its involvement in the degradation of pre-miRNAs, small RNAs and mRNAs. URT1, on the other hand, is primarily localized to the cytoplasm and enriched in P-bodies and stress granules (SG), colocalizing with key decapping and degradation-associated proteins such as Decapping (DCP) and RNA-binding Protein 47 (RBP47) [10,66]. These subcellular localization patterns align with their functional roles in various RNA tailing/degradation pathways. In the nucleolus, NTP10/TRL is functionally validated as the key enzyme responsible for oligo adenylation of rRNA precursors, and thus primarily localized to the nucleolus [74]. Additionally, NTP4 is predominantly localized to the nucleus, where it catalyzes asymmetric tailing of miRNA/miRNA* duplexes to regulate miRNA homeostasis and abundance [58]. However, the subcellular localization of several NTP members (NTP2/5/6/7/8/9) remains to be fully elucidated.

### 4.3. Substrate Specificity Toward Exogenous RNAs: Viral RNA Tailing

The substrate specificity of NTPs extends beyond endogenous RNA to include exogenous RNA, such as viral RNA. In RNA viruses such as Turnip Mosaic Virus (TuMV), HESO1 preferentially targets viral RNAs with extremely short adenine tails (approximately 4 nt), while URT1 tends to modify substrates bearing 10–11 nt adenine tails-a pattern highly consistent with the modification characteristics of endogenous mRNA [43,53]. In the *heso1 urt1* double mutant, TuMV RNA degradation intermediates accumulate markedly, indicating that host TUTases restrict viral replication by uridylating viral RNA and directing it toward degradation pathways [83]. Notably, RNA from different viral sources exhibits markedly distinct and specific uridylation modification profiles, reflecting coevolutionary dynamics between host and pathogen in molecular interactions [83,84].

### 4.4. Beyond Degradation: Non-Degradative Outcomes of RNA Tailing

In the regulation of plant RNA metabolism, 3′-ends tailing mediated by NTPs has long been considered as a degradation signal that triggers RNA degradation. However, increasing evidence indicates that degradation is not the only function in NTPs-mediated tailing. Their functional roles of NTPs are far more diverse and complex than previously recognized, and the underlying molecular regulatory mechanisms remain to be systematical elucidated.

TUT4/7 in mammals precisely regulates the timing of maternal mRNA translation activation through uridylation, suggesting potential analogous mechanisms in plant stress responses and development [88]. This provides cross-species evidence for the involvement of NTPs in translational regulation. Furthermore, the observation that HESO1-mediated uridylation stabilizes the P4RNAs [12], demonstrates the pivotal role of NTP in non-degradative RNA processing pathways. Additionally, in the *hen1* background, URT1-mediated monouridylation modifications, such as the addition of a single uridine tail to miR171a, converts it into a 22-nt isoform, thereby enabling it to trigger phasiRNA production at PHAS gene loci [41,59]. Correspondingly, in a wild-type context, monouridylation of miR1510 mediated by HESO1 in the *Phaseoleae* species enables the specific initiation of phasiRNA biosynthesis from numerous NB-LRR-type disease resistance loci, fine-tuning disease resistance signaling [89]. However, further research is still needed to fully elucidate the non-degradative function of NTP.

In summary, *Arabidopsis* NTP family members achieve specialized roles in RNA metabolism through differentiated substrate recognition mechanisms, including RNA type, terminal nucleotide composition, tail length, subcellular localization and secondary structure. This highly specific substrate selectivity enables NTPs to mediate diverse functions such as degradation labeling, homeostasis maintenance, and quality control, ultimately exerting multi-layered, dynamic regulatory roles in plant development, stress responses, and also antiviral immunity. The ultimate functional outcome of uridylation depends on three key factors: substrate structure, tail characteristics, and spatiotemporal coupling with downstream degradation or processing machinery, demonstrating the complexity and adaptability of the NTP regulatory network. The tail type/length-dependent outcomes across RNA classes are summarized in Table 3.

## 5. Biological Functions of NTP-Mediated RNA Tailing in Plants

Previous studies mainly focused on the molecular functions of NTPs in the regulation of RNA metabolism and homeostasis. However, recent studies increasingly reveal their biological roles in the regulation of plant growth and stress adaptation. Their biological impacts of NTP-mediated RNA tailing largely arise from the fine-tuned modulation of small RNA stability, mRNA turnover, RNA surveillance, and the tailored processing of viral and noncoding RNAs. Below, we summarize the emerging biological functions of NTP-mediated RNA tailing. The corresponding genetic/phenotypic and crop-level evidence is summarized in Table 4.

### 5.1. Regulation of Plant Growth and RNA Homeostasis

The developmental importance of NTP-mediated tailing is most clearly illustrated in *hen1* mutant backgrounds. The *hen1* mutant displayed severe growth defects and poor fertility, which were largely restored in *heso1 hen1* [39,40] and *heso1 urt1 hen1* [42]. Crucially, the extent of the plant phenotype is closely correlated with the small RNA levels in these mutants harboring *hen1* backgrounds, suggesting a direct link between NTP-mediated small RNA tailing and the plant phenotype [39,40,42]. Another key example of the role of NTP in modulating plant growth is its interaction with cytoplasmic RNA degradation factors in the RNA surveillance pathway: the *urt1 xrn4* double mutant fails to initiate leaf formation and inflorescence development, and the *urt1 ski2* double mutant also displays severe growth arrest phenotype [10,53]. *Arabidopsis* terminal nucleotidyl transferases govern secondary siRNA production at distinct steps in these processes [43,68,69]. Additionally, research shows that the *heso1 urt1 ski2* triple mutant exhibits severe leaf chlorosis due to siRNA-mediated silencing of the TKL1 gene in the photosynthesis pathway [69].

### 5.2. Roles in Abiotic Stress Responses

Cross-species analysis has shown that the NTP members are widely involved in plant response to various environmental cues. The promoters of soybean *GmNTP* genes are enriched with numerous stress response elements, including 133 light response elements (such as G-box, GT1, TCCC, TCT, etc.) and various hormone (such as abscisic acid (ABA), methyl jasmonate, salicylic acid, etc.) response elements, suggesting a potential role of NTP in light signaling and hormone pathways [46]. Consistently, expression profiling show that multiple *OsNTP* genes in rice respond to abiotic stresses [47], while different NTP in maize exhibit differential expression patterns under drought and salt stress, highlighting the conservation and diversity of their stress response mechanisms [44]. In wheat, multiple *TaNTP* genes show pronounced transcriptional responses to abiotic stresses, especially under combined heat–drought conditions, and TaNTP6A/B/D are strongly induced by salt treatment. These findings support the conserved involvement of NTP family members in stress-responsive RNA regulatory programs [49]. Moreover, natural variation within the NTP family is closely linked to important agronomic traits. In rice, a single nucleotide polymorphism (SNP) in the *HESO1* homolog gene *LOC_Os01g62790* causes a valine/isoleucine substitution. Varieties carrying haplotype A (valine) exhibit significantly earlier heading than those carrying haplotype B (isoleucine), and transgenic experiments confirm that haplotype B Nipponbare plants show markedly delayed heading [45]. In wheat, haplotype–trait associations suggest TaNTP6A/B/D may contribute to grain development and yield potential [49]. Notably, NTPs also participate in plant immune regulation through viral RNA modification. Analysis of 47 positive-strand RNA plant viruses revealed uridylation modifications (0.2–90%) in all viral RNAs, where HESO1 preferentially targets TuMV RNA with shorter poly(A) tails (median length 4 nt), while URT1 tends to modify viral RNA with 10–11 nt polyadenylation tails. This differential modification pattern may guide viral RNA degradation, constituting a crucial component of plant antiviral mechanisms [83,84].

### 5.3. Functional Differentiation of the NTP Family Across Species

The molecular functions of several plant NTPs have been elucidated through a combination of genetic and biochemical approaches. However, their biological roles in plant growth and development have received relatively little attention. Although the NTP family is conserved in plants, functional diversification still exists among different species (Figure 1). In rice, *OsNTP* genes exhibit specific induction under various stress treatments, including salt, drought, heat, cold, or abscisic acid (ABA). For instance, *OsNTP5/6/7/8* are induced by salt stress, *OsNTP1/2/4/9/13* are upregulated under drought conditions, *OsNTP4/5/6* are ABA-induced, while *OsNTP3/4/5/12/13* are downregulated during cold treatment [47]. In maize, the response of *ZmNTP* genes to stress is more complex. *ZmNTP1/5/6/9/10/11/12/16/17/18/19/20/21/22* are significantly downregulated under aboveground drought stress, while only ZmNTP23 and ZmNTP24 are upregulated. In contrast, in the roots, *ZmNTP2/3/4/8/9/17/20/22/23/24* are induced [44]. In soybean, *GmNTP10* and *GmNTP14* (*HESO1* homologs) show high expression in root nodules, suggesting they may influence nitrogen fixation by regulating miRNA processing or degradation within nodules. Additionally, *GmNTP5* and *GmNTP16* exhibit unique expression patterns: *GmNTP5* is expressed almost exclusively during early seed development, while *GmNTP16* is undetectable in any tissue except reproductive tissues, suggesting these genes may possess specialized functions [46]. Wheat (*Triticum aestivum*) further highlights lineage-specific diversification of NTP regulation through strong tissue- and stress-combination dependence. Several TaNTPs show contrasting responses between roots and leaves under drought, heat, and especially combined heat–drought stress, for example, TaNTP6B/D and TaNTP7A are induced in roots by drought/heat or their combination, whereas other members (e.g., TaNTP7A/B and TaNTP11B) are repressed under drought/heat-related conditions. In developing grains, distinct TaNTP members (e.g., TaNTP4B, TaNTP6D, TaNTP7B/D, TaNTP10A) respond to drought and/or heat–drought stress, further underscoring organ- and stage-specific specialization [49].

## 6. Perspectives and Future Directions

Although significant progress has been made in characterizing the enzymatic functions, substrate preferences, and biological roles of plant nucleotidyltransferases (NTPs), our current understanding of RNA tailing is still fragmented. Recent discoveries have greatly broadened the conceptual landscape, demonstrating that NTPs can stabilize or destabilize RNAs, act across multiple RNA classes, and integrate into diverse RNA metabolism pathways, yet these findings also reveal mechanistic, structural, and physiological complexities that remain unresolved. To advance toward an integrated framework of NTP-mediated RNA regulation, several major scientific questions need to be addressed.

### 6.1. Mechanistic Principles of Degradative Versus Non-Degradative Tailing

Research on NTPs is shifting from the classical degradation-driven perspective toward a new framework in which 3′-end tail modifications produce diverse outcomes across RNA classes under different conditions [3]. Pioneering work on HESO1 and URT1 has established uridylation as a dominant mechanism governing the homeostasis of miRNAs/siRNAs and mRNAs. In some biological pathways, mono-/di-uridylation stabilize the processing of precursors, whereas oligouridylation is more commonly correlates with RNA degradation. Beyond RNA type and terminal structure, tail identity and tail length add additional regulatory complexity: mono-/di-uridylation is often associated with processing/functional reprogramming, whereas oligo-uridylation frequently correlates with decay engagement, depending on the RNA class and context [12,39,40,41,53]. A representative case is miR171a, where URT1-mediated monouridylation generates a 22-nt isoform capable of triggering phasiRNA biogenesis [41,59]. However, the substrate profiles, tail types (e.g., A-tail, U-tail, or mixed tails), and physiological functions of other NTP members remain largely uncharacterized. Furthermore, current phenotypic analyses are often confined to the molecular level, failing to establish clear causal relationships between NTP activity and vital biological processes, such as growth, development, and stress adaptation.

Although NTP-mediated non-templated tailing is frequently framed as a quality-control mechanism for precursor RNAs, the consequences of pre-miRNA tailing are not fully consistent across studies. In some contexts, limited tailing may facilitate precursor processing or “repair” misprocessed ends, whereas in others, tailing appears to promote the clearance of defective precursors. These divergent outcomes likely reflect differences in RNA-end features, misprocessing severity, the HEN1-dependent 2′-O-methylation status, subcellular compartmentation (nucleus versus cytoplasm), and the degree to which tailing is coupled to downstream ribonucleases and decay pathways. Importantly, whether these outcome differences represent true species-specific divergence or primarily context-dependent regulation remains insufficiently explored and should be addressed through direct comparative genetic and biochemical analyses across representative crops and model species.

### 6.2. Expanding the Diversity of RNA Substrates and Tailing Pathways

Beyond the canonical uridylation process, additional layers of complexity in RNA tailing complexity have emerged. For example, TRL/NTP10 reveals an adenylation-based paradigm for RNA quality control. This mechanism involves the oligoadenylation of pre-rRNAs, which subsequently directs them for degradation by the exosome [74]. However, it remains unclear whether pre-rRNA uridylation operates in parallel, and which enzymes mediate this process in plants [74]. NTP4 adds an additional dimension of complexity by acting in the nucleus on miRNA/miRNA* duplexes, where asymmetric end modification can fine-tune strand selection and mature miRNA output [58]. The diversity of tailing types mediated by NTPs reflects the multifaceted roles they play in RNA regulation. Currently, a core scientific question persists: how do NTPs distinguish between “degradative” and “non-degradative” tailing? In other words, the molecular mechanisms underlying their substrate selection and tail length control remain unclear. Structural studies of URT1, including crystal structures and mechanistic analyses, have provided a foundation for understanding plant RNA uridylation [86,87]. These studies have established a framework for understanding how the NTase catalytic core relates to nucleotide donor selection and tail-length regulation, particularly by constraining how the RNA 3′ end and incoming nucleotide are positioned in the active site [86,87]. They also help explain URT1’s preference for specific RNA-end states (e.g., short poly (A) tails) and the bias toward short versus longer U-tails under different contexts [53,86,87]. In contrast, comparable high-resolution structural information for HESO1-like enzymes remains limited, highlighting an important gap in the field. Building on this, an important future direction is to reconstruct representative NTP–RNA–cofactor complexes to unveil the molecular mechanisms of nucleotide donor selection and tail length control at the atomic level, and ultimately to identify the terminal sequences or structural features of the RNA that determine the outcome of tailing, whether it favors stabilization, maturation, or clearance. Such advances will be crucial for elucidating the substrate selectivity and functional differentiation of NTPs.

### 6.3. Structural Disorder, Phase Separation, and Spatial Regulation of NTP Activity

Many members of the NTP family are notably rich in IDRs, and mounting evidence suggests that these IDRs may confer multivalent interactions and phase-separation, enabling NTPs to localize to membraneless organelles such as P-bodies. IDRs provide multivalent interaction platforms that may tune substrate access and local enzyme concentration within condensates (e.g., P-bodies and stress granules), thereby influencing RNA tailing outcomes in a context-dependent manner. For instance, the N terminus of URT1 contains a large IDR, enriched in low-complexity residues and multiple conserved short linear motifs, endowing the protein with multivalent interactions and phase-separation potential [53]. Imaging and interactome data show that, in addition to a diffuse cytosolic pool, URT1 concentrates in P-bodies and can enter stress granules, where it co-localizes and interacts with decay factors such as DCP5 and the CCR4–NOT complex. Functionally, URT1 preferentially uridylates short-tailed mRNAs to promote decapping and 5′→3′ exonucleolytic decay, underscoring its central role in the cytoplasmic tailing–decay pathway [8,10,53]. Within P-bodies, NTP8 has been shown to co-localize with UPF1. a key regulatory factor of the nonsense-mediated mRNA decay (NMD) pathway [85]. However, reliable subcellular localization maps for all ten members of the *Arabidopsis* NTP family remain incomplete, limiting our understanding of their specific functions. Of particular interest is whether the potential IDRs within these proteins regulate their subcellular localization and function by mediating processes such as phase separation. Therefore, a systematical investigation into the localization of each family member is crucial for fully revealing the biological roles of the NTP family.

### 6.4. Physiological Roles of NTPs and Their Links to Plant Fitness

In the model plant *Arabidopsis thaliana*, the NTP-mediated phenotype has been observed in backgrounds of *hen1*, *xrn4* or *ski2* [5,39,53,69]. However, no obvious phenotypic changes have been observed in *ntp* single or multiple mutants. To date, stress-related phenotypes of *ntp* single mutants have been subtle or context dependent, and systematic stress assays across family members remain limited. Research on NTPs in crops has primarily focused on macroscopic phenotypes and gene expression levels, lacking deeper depth mechanistic insights. This presents a critical, unanswered question: can the NTP-mediated RNA stability regulation mechanism mediated by NTPs be leveraged to improve crop resilience, yield, or quality? For example, recent association analyses in wheat suggest that TaNTP6A/B/D haplotypes correlate with TKW, highlighting NTP loci as potential targets for yield-related trait optimization [49]. Nevertheless, establishing causality will require direct functional validation. For instance, targeted editing of specific NTP genes could offer a path for precisely modulating the stability and expression of key RNAs associated with vital agronomic traits.

In summary, the functions of the NTP family extend well beyond RNA degradation. They are central to multiple critical processes, including RNA stabilization, processing and maturation, translational control, and interactions with biotic and abiotic environments. In several crops, NTP family members show stress-responsive transcriptional changes and stress/hormone-related cis-element enrichment; however, much of the current evidence remains correlative, and direct causal links have yet to be established. Because stress treatments broadly remodel transcriptional programs and RNA metabolism, expression-based associations alone may generate false positives and fail to establish causal roles for NTPs in stress tolerance. Future research should integrate cutting-edge technologies—such as multi-omics, single-molecule imaging, and gene editing—to systematically elucidate the functional diversity and regulatory networks of NTPs at molecular, cellular, and organismal levels. This approach will not only deepen our understanding of RNA biology but also offer powerful tools and strategies for crop genetic improvement.

## Figures and Tables

**Table 1:** Comparative overview of NTP gene families across *Arabidopsis* and major crops.

Species	NTP Members (n)	Evolutionary Features	Functional Validation Available	Reference
*Arabidopsis thaliana*	10	Reference set	Yes (multiple members)	[43]
Rice (*Oryza sativa*)	13	Moderate expansion	Yes (selected loci)	[45,47]
Maize (*Zea mays*)	24	Strong expansion	Limited	[44]
Soybean (*Glycine max*)	16	WGD retention	Limited	[46]
Wheat (*Triticum aestivum*, hexaploid)	34	Polyploid homeolog retention	Partial (association; limited functional tests)	[49]

**Table 3:** Tail type/length-dependent outcomes (“tail code”) across RNA classes in plants.

RNA Class	Typical Tail Type	Main NTP(s)	Context/Trigger	Primary Outcome	Reference
Mature miRNA	U-tails; mono/di vs. oligo (context-dependent)	HESO1 (major); URT1 (backup)	HEN1 loss/unmethylated miRNAs; miRNA turnover	Mostly degradation/clearance; but length-dependent rewiring can occur	[39,40,41]
siRNA (multiple size classes)	U-tails (enhanced when methylation compromised)	HESO1, URT1	Loss of 2′-O-methylation protection; enzyme perturbation	Often promotes turnover/quality control	[39,42]
pre-miRNA	U- and/or C-tailing; variable length	HESO1; NTP6/7	Mis-processing/abnormal ends	Processing vs. degradation	[11]
RISC-cleavage fragments (5′)	U-tailing	HESO1 (major), URT1	miRNA-guided cleavage; RISC recycling pathway	Decay/clearance	[48,65,66]
mRNA (poly(A)-associated)	U addition at poly(A) tail end (often short U-tract)	URT1 (major)	mRNA turnover; deadenylation state	Promotes proper turnover; can prevent excessive deadenylation	[9,10,53]
Pol IV-derived RNAs (P4RNA)	mono/di-U and mixed tails reported	HESO1	RdDM pathway; Pol IV/RDR2-derived transcripts	Enhance stability	[12]
pre-rRNA (18S/5.8S precursors)	A-tailing	TRL (NTP10)	rRNA quality control	Exosome recruitment → targeted turnover	[74]
Viral RNAs (+ssRNA viruses)	U-tailing frequency variable	HESO1 (noted preference); URT1 may contribute	Antiviral RNA surveillance	Degradation; possibly stability effects in some contexts	[83,84]

**Table 4:** Genetic and functional evidence linking NTPs to agronomic traits and stress/antiviral performance in crops.

Species	Gene/Locus	Evidence Type	Trait/Phenotype	Reference
*Arabidopsis*	*heso1 hen1*	genetics (double mutant)	Rescues hen1 growth defects and poor fertility	[39,40]
	*heso1 urt1 hen1*	genetics (triple mutant)	Stronger rescue of hen1-associated defects	[69]
	*urt1 xrn4*	genetics (double mutant)	Failure to initiate leaf formation and inflorescence development	[10,53]
	*urt1 ski2*	genetics (double mutant)	Severe growth arrest	[53]
	*heso1 urt1 ski2*	genetics (triple mutant)	Severe leaf chlorosis	[69]
Rice	*LOC_Os01g62790* (HESO1 homolog)	GWAS + transgenic validation	Heading time	[45]
Wheat	TaNTP6A/B/D	haplotype association and expression	Thousand-kernel weight (TKW); grain-enriched expression	[49]
	TaNTPs (multiple)	Expression under stress	Heat–drought; salt induction (TaNTP6 triad strong)	[49]
Maize	ZmNTP family (multiple members)	Expression under stress	Drought and salt response transcriptional changes	[44]
Soybean	GmNTP family	Promoter cis-element enrichment and expression profiling	Light/hormone/stress responsiveness	[46]
(Cross-plant viruses)	HESO1/URT1 (plant antiviral context)	Viral RNA tailing surveys and mechanistic inference	Antiviral immunity relevance	[83,84]

## Data Availability

Data are contained within the article and Supplementary Materials.

## Supplementary Materials

The following supporting information can be downloaded at: https://www.mdpi.com/article/10.3390/plants15060925/s1. Table S1: NTP family members of *Arabidopsis*, rice, maize, soybean and wheat including gene name, gene ID and protein sequence.

## Abbreviations

The following abbreviations are used in this manuscript:

ABA	Abscisic acid
AGO/AGO1	ARGONAUTE/ARGONAUTE1
DCL	DICER-LIKE
DCP5	Decapping protein 5
dsRNA	Double-stranded RNA
GWAS	Genome-wide association study
HEN1	HUA ENHANCER 1
HESO1	HEN1 SUPPRESSOR 1
IDR(s)	Intrinsically disordered region(s)
mRNA	Messenger RNA
NMD	Nonsense-mediated mRNA decay
NTP(s)	Nucleotidyltransferase protein(s)
P4RNA	Polymerase IV RNA
PTGS	Post-transcriptional gene silencing
RdDM	RNA-directed DNA methylation
siRNA(s)	Small interfering RNA(s)
sRNA(s)	Small RNA(s)
SNP	Single nucleotide polymorphism
TKW	Thousand-kernel weight
TNT(s)	Terminal nucleotide transferase(s)
TUT/TUTase	Terminal uridylyltransferase
URT1	URIDYLYL TRANSFERASE 1
WGD	Whole-genome duplication
